# A colorectal cancer missed by colon capsule endoscopy: a case report

**DOI:** 10.1186/s12876-022-02332-8

**Published:** 2022-05-21

**Authors:** C. MacLeod, R. Oliphant, J. G. Docherty, A. J. M. Watson

**Affiliations:** Department of Surgery, Raigmore Hospital, Inverness, UK

**Keywords:** Colon capsule endoscopy, Colorectal cancer, Colonoscopy

## Abstract

**Background:**

Colon capsule endoscopy (CCE) is a non-invasive alternative to colonoscopy. The reported sensitivity and specificity of CCE for the detection of clinically significant colonic neoplasia is high. To date, there have been no reported cases of colorectal cancer (CRC) missed by CCE which were located in segments adequately visualised by the capsule.

**Case presentation:**

We present the case of a 71-year-old female, who underwent CCE for new lower gastrointestinal symptoms. The CCE reported 17 polyps (largest size 10 mm) and angiodysplasia. A 40 mm caecal pole tumour, not detected by the CCE, was identified at follow up colonoscopy. Surgical resection was performed, and the pathology sample was reported as moderately differentiated adenocarcinoma, pT2 pN0 (0/19) M0, with no evidence of EMVI. The patient made an uneventful recovery. The caecal pole tumour was not definitively identified on retrospective review of the CCE images.

**Conclusion:**

To date, this is the first published case of a CRC missed entirely by CCE. Further research is required to allow calculation of the post CCE interval CRC rate to allow comparison with colonoscopy and CT colonogram.

**Supplementary Information:**

The online version contains supplementary material available at 10.1186/s12876-022-02332-8.

## Background

Colon capsule endoscopy (CCE) is a non-invasive alternative to colonoscopy. A recently published systematic review and meta-analysis reported CCE to have high accuracy for the detection colonic neoplasia compared to colonoscopy [1]. Furthermore, it was also reported that CCE did not miss any colorectal cancer (CRC) located in a colonic segment adequately visualised by CCE. Significant lesions “missed” by CCE should be investigated, and reported where appropriate, to improve our understanding of this technology. We present the case of a patient who underwent CCE which entirely missed a colorectal cancer subsequently identified at colonoscopy.

## Case presentation

A 71-year-old female was referred to the colorectal service with bleeding per rectum and loose bowel motions. Her past medical history included colonic polyps for which she had last undergone colonoscopy 19 years before the current investigations. Routine blood tests showed a haemoglobin count of 113 g/L with a normal mean cell volume. Her faecal immunochemical test result was > 400 µgHb/g.

**Fig. 1:**
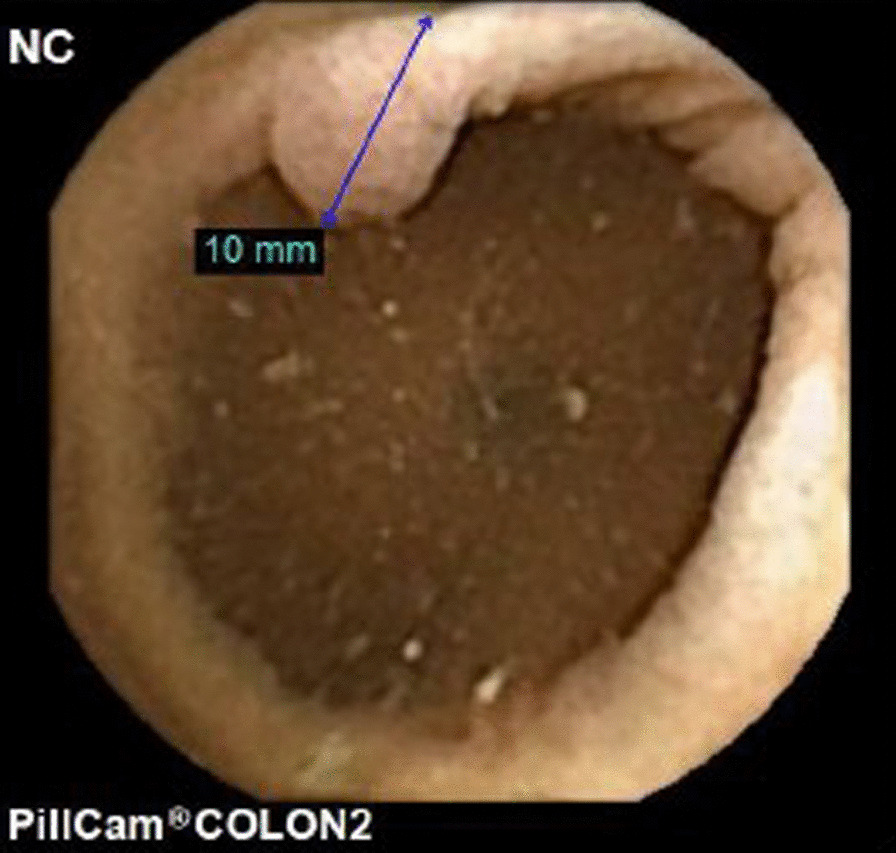
10 mm polyp in left colon seen on CCE

**Fig. 2:**
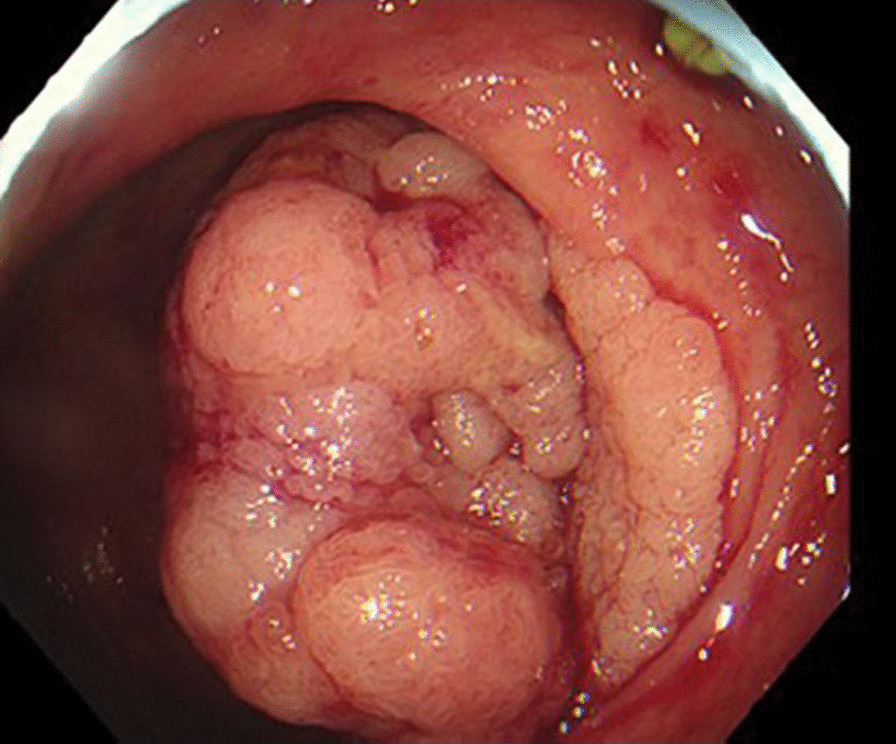
Caecal tumour found at colonoscopy

The patient underwent a colon capsule endoscopy (CCE) which was reported by a consultant gastroenterologist with experience of reading over 200 CCE procedures. The bowel preparation regimen and dietary recommendations, and protocol used for the procedure are described in “Appendices 1 and 2”. The CCE reader reported that all segments of the colon and rectum were visualised, with good bowel preparation throughout. The transit times for the capsule, in minutes, from mouth to cecum, cecum to hepatic flexure, hepatic to splenic flexure was 191, 96 and 6 respectively. The total colon passage time was 471 min. 17 polyps were reported, the largest polyp was 10 mm, located in the left colon (Fig. 1). 5 of the 17 polyps reported were in the right colon, size range 5-7 mm. Angiodysplasia was also detected and was thought to be the cause of the rectal bleeding. The patient subsequently underwent a routine colonoscopy, for polypectomy. The colonoscopy was performed with the CCE report available and revealed a 40 mm polypoid tumour with a malignant appearance in the caecal pole just proximal to the ileocaecal valve (Fig. 2). 2 further polyps were identified at colonoscopy, a 5 mm polyp at the hepatic flexure and a 3 mm polyp in the sigmoid colon. Despite the macroscopic concern of invasion within a large villous polyp the histopathology from the biopsies of the caecal lesion were reported as tubulovillous adenoma with low grade dysplasia.

A staging CT scan of the thorax, abdomen and pelvis identified the caecal pole mass with no evidence of local or metastatic disease. Given the size and appearance of the tumour, the patient underwent a laparoscopic right hemicolectomy with primary anastomosis. Her recovery was uneventful, and she was discharged on the 4th post-operative day. The right hemicolectomy pathology specimen was reported as a moderately differentiated adenocarcinoma, pT2 pN0 (0/19) M0 with no EMVI, R0 resection, stage 1, Dukes’ A. The tumour was KRAS variant (Gly12Val), BRAF non-variant and MSI was detected.

Subsequent review of the CCE recording, and the colonoscopy images, was carried out. Despite frame-by-frame analysis, the caecal pole tumour could not be definitively identified on the CCE images by the original CCE reader, or by an additional CCE reader with experience of reading over 500 CCE procedures (Additional file 1). On re-review of the polyp images, there was no change to the number of polyps reported, although some were noted to be subtle. While one polyp was found at colonoscopy at the hepatic flexure, none of the remaining 4 polyps in the right colon reported by CCE were found at colonoscopy or on examination of the pathology specimen.

## Discussion and conclusion

To our knowledge, this is the first published case of colorectal cancer (CRC) missed by CCE, where the CRC was in a colonic segment reported as adequately visualised by CCE, and the cancer was not visible on subsequent review. The accuracy of CCE for the detection of CRC has been reported by a recently published meta-analysis and systematic review of 12 CCE studies involving 2199 patients [1]. Kjølhede reports that out of 54 CRC identified by colonoscopy across the studies, 44 were correctly identified by CCE with the remainder either present in segments not visualised by the procedure (n = 8), size misjudged (n = 1) or detected on second review (n = 1). Given the position of CCE as an alternative to colonoscopy and CT colonogram (CTC), it is important to consider the interval CRC rate of all available investigations.

The post-colonoscopy colorectal cancer rate (PCCRC) has been examined in a large population-based cohort study [2]. The overall adjusted PCCRC 3-year rate reported by Burr was 7.4%. A lower rate was found for colonoscopies performed under the National Health Service (NHS) England bowel cancer screening programme (3.6%) and a higher rate found for those performed by independent, non-NHS providers (9.6%) and in those performed for symptomatic indication. Comparatively, for CTC, the post-imaging colorectal cancer rate at 34 months is 4.4% [3]. There is no published post CCE interval CRC rate.

The limitations of CCE to visualise other areas of the colon should also be considered following this case. The caecal pole has been highlighted as an area of concern where significant pathology could be missed by CCE. Further “blind spots” for CCE may become apparent and should be discussed transparently as they are discovered.

The follow up colonoscopy for this patient was triggered by multiple polyps reported by CCE. However, many of these polyps were not found at colonoscopy, and none were identified in the right hemicolectomy pathology specimen. The specificity of CCE is an accepted weakness of the test and clinicians should be aware that CCE may “over” report the number polyps due to the nature of the test and dynamic nature of the colonic mucosa [4].

It is important that CCE continues to be evaluated, particularly as CCE availability increases in the NHS [5–7]. Efforts should be made to capture outcome data to allow the post CCE interval CRC rate to be reported. Longitudinal follow up or record linkage to cancer registries will be crucial to achieving this.

### Supplementary Information

**Additional file 1.** Colon capsule endoscopy procedure video of capsule passing into caecum.

## Data Availability

Not applicable.

## Appendix 1

**Table:**

Bowel preparation regimen and dietary recommendations for CCE procedure		
Day	Preparation	Dietary recommendations
3 Days before procedure	1 Sachet of Macrogol 3350 in 125 ml of water morning and evening	Normal diet
2 Days before procedure	1 Sachet of Macrogol 3350 in 125 ml of water morning and evening	Low residue diet
1 Day before procedure	2 Litres of Polyethylene glycol solution taken over 2 h in the evening	Clear liquid diet
Day of procedure	2 Litres of Polyethylene glycol solution taken over 2 h in the morning	Clear liquid diet
	Metoclopramide hydrochloride 10 mg tablet taken immediately after the colon capsule is swallowed	Return to normal diet 6 h after capsule ingestion or once excreted
	1 Litre of Sodium picosulfate solution taken as indicated by the three data recorder signals	
	1 Bisacodyl suppository (optional) to be used if the capsule has not been excreted 10 h after swallowing the capsule	

## Appendix 2

### CCE procedure protocol

1. Patients agreeing to participate are screened by a nurse reviewing their electronic healthcare records.
2. The first telephone consultation is carried out by a nurse with the patient to explain the procedure in detail, confirm consent to continue with the procedure and arrange test date.
3. The bowel preparation with instructions is sent to patients’ home, regimen detailed in “Appendix 1”.
4. A second telephone consultation is carried out by a nurse with the patient to provide additional support for bowel preparation consumption.
5. The patient attends the CCE procedure, which is carried out by a trained nurse.
6. Pre procedure checks are carried out to ensure the patient is safe to continue with the procedure and that the bowel preparation has been adequate. If bowel cleanliness is inadequate, then one sachet of picolax in one litre of water is administered.
7. The belt and recorder are fitted, then the capsule is swallowed by the patient.
8. The booster medication is provided to the patient with further instructions.
9. The patient returns home to complete the procedure returning the belt and recorder the following day.

## Abbreviations

CCE: Colon capsule endoscopy
CRC: Colorectal cancer
CTC: CT colonogram
NHS: National Health Service
PCCRC: Post-colonoscopy colorectal cancer rate

## References

[1] Kjolhede T, Olholm AM, Kaalby L, Kidholm K, Qvist N, Baatrup G (2020). Diagnostic accuracy of capsule endoscopy compared with colonoscopy for polyp detection: systematic review and meta-analyses. Endoscopy.

[2] Burr NE, Derbyshire E, Taylor J, Whalley S, Subramanian V, Finan PJ, et al. Variation in post-colonoscopy colorectal cancer across colonoscopy providers in English National Health Service: population based cohort study. BMJ. 2019;367. 10.1136/bmj.l6090 PMC6849511 31722875

[3] Obaro AE, Plumb AA, Fanshawe TR, Torres US, Baldwin-Cleland R, Taylor SA (2018). Post-imaging colorectal cancer or interval cancer rates after CT colonography: a systematic review and meta-analysis. Lancet Gastroenterol Hepatol.

[4] González-Suárez B, Pagés M, Araujo IK, Romero C, Rodríguez De Miguel C, Ayuso JR (2020). Colon capsule endoscopy versus CT colonography in FIT-positive colorectal cancer screening subjects: a prospective randomised trial—the VICOCA study. BMC Med.

[5] NHS England » NHS rolls out capsule cameras to test for cancer. https://www.england.nhs.uk/2021/03/nhs-rolls-out-capsule-cameras-to-test-for-cancer/.

[6] Pill camera procedure launched in fight against bowel cancer—BBC News. https://www.bbc.co.uk/news/uk-scotland-tayside-central-55130655.

[7] NHSGGC: New technology for patients being tested for bowel cancer. https://www.nhsggc.org.uk/about-us/media-centre/news/2020/12/new-technology-for-patients-being-tested-for-bowel-cancer/#.

